# College and Hospital Notes

**Published:** 1911-06

**Authors:** 


### COLLEGE AND HOSPITAL NOTES

The new McAlpine hotel in course of construction at Broadway and Thirty-fourth street, New York, when complete will have within its walls a completely equipped modern hospital section where any character of case from the most trivial to the most serious can be cared for as readily as in the larger, modern hospitals. The operating room will be on the twenty-third floor and there will be accommodations for twelve patients. The location of the rooms makes it certain that patients will not be disturbed by street noises.

---

Health Commissioner Fronczak has made a special report on the Ernest Wende Hospital to the Buffalo Board of Aldermen which he based upon the annual report of the superintendent, Dr. Walter S. Goodale. The report recites that the institution has been brought to its present state of efficiency by an expenditure of $22,000, the abandoned school at Broadway and Spring street having been remodeled and equipped with the most modern appliances. Dr. Goodale estimates the value of the hospital to be $91,963. There were 992 patients cared for last year.

---

Mayor Fuhrmann has vetoed the Aldermanic resolution directing the purchase of the West farm as a site for a city general hospital at a cost of approximately $225,000. The Mayor suggested that it would be more economic to secure the present Erie county farm for the hospital site.

---

There will be a reunion of the graduates in medicine of McGill University at Montreal on June 5 and 6. During the reunion the new buildings will be formally opened by the Governor General. The Canadian Medical Association will meet in Montreal during the week of the reunion. The program for the reunion is as follows:

Monday, June 5th—Informal luncheon at the McGill Union by members of teaching staff of the medical department to the graduates in medicine. Afternoon—Convocation in Royal Victoria college. Evening—Opening of new building. Conversazione given by the governors of the University.

Tuesday, June 6th—Morning and Afternoon—Clinics and demonstrations in hospitals and laboratories; private entertainments. Evening—Banquet tendered by the members of teaching staff of the Medical Department to the graduates in medicine.

---

The delegates of the United States to the International Committee for Post-Graduate Medical Education will maintain a bureau of information on medical education, particularly post-graduate medical education. All available information on this subject will be kept on file for the benefit of those who inquire personally or by mail about the educational facilities in the different medical centers of the world. This bureau will be located at 303 East 20th Street, New York City, and will bear the name of: American bureau of information of the international committee for post-graduate medical education. All communications should be addressed to "Medical Information Bureau, 303 East 20th Street, New York City." Communications requiring answer must be accompanied by stamped envelope. The delegates of the United States are: Dr. G. N. Miller, New York City; Dr. Ed. Quintard, New York City; Dr. W. S. Thayer, Baltimore, Maryland; Brigadier-General, G. H. Torney, Surgeon-General of the United States Army, Washington; Dr. C. F. Stokes, Surgeon-General of the United States Navy, Washington; Secretary for the United States of America: Dr. L. Kast, New York City.

---

The Day Camp for tuberculosis will open for its fourth season on May 31 this year. Accommodations will be provided for from thirty to thirty-five patients, day and night, and for as many additional day patients as can be cared for. The purpose of the camp is to care for tuberculosis patients who are unable to pay for sanitorium treatment. Physicians having suitable cases under their care and who wish to secure admission for the patient to the Day Camp are requested to send the patient to the tuberculosis dispensary, No. 165 Swan street, between the hours of 10 o'clock and noon every day except Sundays and holidays.

---

THE Twin City Hospital Association, of Tonawanda, N. Y., at a meeting held April 23, elected Honorable James P. McKenzie, former state senator, president and Frederick Robertson, treasurer. The association will raise funds for the erection of a hospital.

---

DR. EVA FOWLER, Dr. Rose Donk and Dr. Frances Flood have been appointed interne physicians at the Erie County Hospital after competitive examination. These appointments are the first for women physicians to be made in Buffalo.

---

DR. CARRINGTON, of the State Charities Aid Association, visited Lockport on May 16 and inspected proposed sites for the tuberculosis hospital which Niagara county will erect. The sites submitted are located at Lockport, Niagara Falls and Sanborn. Dr. J. A. Lewis, president of the Lockport tuberculosis committee, is taking an active part in the work of relief in Niagara county.

---

JUSTICE WHEELER in special term of the Supreme Court granted an order on May 18 permitting the consolidation of the Buffalo Hahnemann and Buffalo Homeopathic hospitals under the title of the Buffalo Homeopathic hospital.

The new hospital is located at Linwood and Lafayette avenues. Under the terms of the consolidation the new Buffalo Homeopathic hospital will occupy these quarters, and the provision is made that the attending medical and surgical staff of the hospital shall be members of the homeopathic school. The assets and liabilities of each of the hospitals are to become the joint assets and liabilities of the new institution.

The petition presented to the court states that there are to be eighteen directors and that the directors, for the first year, are to be the following: William H. Crosby, Frederick C. Gratwick, Henry E. Montgomery, Edward J. Barcalo, Frank L. Bapst, Herbert E. Crouch, Marcus M. Darr, James H. Dyett, Edward A. Eisele, Eugene L. Falk, Chauncey J. Hamlin, Clark L. Ingham, Andrew Langdon, William E. Robertson, Walter H.

Schoellkopf, Harry Yates, Sidney McDougall and Walter P. Cooke.

---

THE Erie County supervisors committee on county home and hospital has reported in favor of entirely separating the tuberculosis hospital from the home and placing it under the management of a board of five trustees two of whom shall be physicians. There will be a public hearing on the question.

---

THE board of managers of the Children's Hospital at the May meeting received reports of a most encouraging character regarding the work of the hospital. The commencement exercises for the graduating class of nurses will be held in June. The graduates are: Maude Pierce, Mae Clark, Hazel Hotson, Mary Earon, Rose Barry, Harriet Rumsey, Rose Gibson, Eva Hamilton, Anna Van Valkenburg.

Two new verandas are being built at the rear for the out-of-doors care of convalescents. Reports of the narcissus sale showed it to have been the most successful ever held. Visitors for the month are Mrs. Clinton R. Wyckoff, Mrs. Jesse Dann, Mrs. William Warren Smith, and Mrs. Lester Wheeler.

---

THE musical spectacle Summer Idylles, presented at Convention Hall on May 26 and 27 by the Ladies' Circle of the German Hospital was a splendid success and netted a considerable sum for the hospital.

---

THE programme for the commencement exercises of the Medical Department of the University of Buffalo, May 30, 31 and June 1, are as follows:

Tuesday, May 30: Alumni Meeting, 8 P.M., at the college, followed by a collation.

Wednesday, May 31, Morning Clinics: 9-10, Obstetrics and gynecology—Drs. Van Peyma, Mann, King and Goldsborough. 10-11, Surgery—Drs. Park, Eugene Smith, Clinton and McGuire. 11:30, Medical—Drs. Stockton and Cary and their assistants. 1-2, Luncheon.

Afternoon Clinics: 2-3:15, Eye clinic by Dr. Howe and assistants. 3:15-4, Ear, nose and throat clinic by Dr. Hinkel and assistants. 4-4:45, Neurological clinic by Dr. Putnam and assistants. 4:45-5:30, Pediatrics clinic by Dr. Snow and assistants. 5:30-6, Dermatology by Dr. Wende and assistants.

Wednesday night will be devoted to class reunion and fraternity night.

June 1, Commencement exercises at the Teck Theatre.

The committee has decided, in view of the unavoidable absence of Dr. Cary to omit the alumni dinner in his honor which had been planned for Wednesday night.

